# Subclinical involvement of the liver is associated with prognosis in treatment naïve cancer patients

**DOI:** 10.18632/oncotarget.17131

**Published:** 2017-04-16

**Authors:** Noemi Pavo, Markus Raderer, Georg Goliasch, Raphael Wurm, Guido Strunk, Anna Cho, Johannes F. Novak, Heinz Gisslinger, Günther G. Steger, Michael Hejna, Wolfgang Köstler, Sabine Zöchbauer-Müller, Christine Marosi, Gabriela Kornek, Leo Auerbach, Sven Thorben Schneider, Bernhard Parschalk, Werner Scheithauer, Robert Pirker, Barbara Kiesewetter, Richard Pacher, Christoph Zielinski, Martin Hülsmann

**Affiliations:** ^1^ Department of Internal Medicine II, Division of Cardiology, Medical University of Vienna, Vienna, Austria; ^2^ Department of Internal Medicine I, Division of Oncology and Hematology, Medical University of Vienna, Vienna, Austria; ^3^ Complexity Research, Vienna, Austria; ^4^ Department of Gynaecology, Medical University of Vienna, Vienna, Austria; ^5^ Department of Otorhinolaryngology - Head and Neck Surgery, Medical University of Vienna, Vienna, Austria

**Keywords:** cancer, liver, biomarker, inflammation, prognosis

## Abstract

**Background:**

Routinely tested liver biomarkers as alanine aminotransferase (ALT), aspartate aminotransferase (AST), γ-glutamyltransferase (GGT), butyryl-cholinesterase (BChE), albumin and bilirubin are altered in distinct malignancies and hepatic metastases. This study aimed to investigate whether all liver parameters have the ability to predict long-term mortality in treatment naïve cancer patients but without a malignant hepatic involvement.

**Methods:**

We prospectively enrolled 555 consecutive patients with primary diagnosis of cancer without prior anticancer therapy. BChE, albumin, AST, ALT, GGT and bilirubin as well as the inflammatory makers C-reactive protein (CRP), serum amyloid A (SAA) and interleukin-6 (IL-6) were determined. All-cause mortality was defined as primary endpoint.

**Results:**

During a median follow-up of 25 (IQR16-31) months 186 (34%) patients died. All liver parameters were significantly associated with all-cause mortality (p < 0.001 for all). However, for patients without a malignant primary or secondary hepatic involvement (82%) only the functional parameters BChE and albumin remained significantly associated with the primary endpoint (crude HR per 1-IQR increase 0.61, 95%CI:0.49-0.77; *p* < 0.001 for BChE and 0.58, 95%CI:0.47-0.70; *p* < 0.001 for albumin). This e ect was persistent after multivariate adjustment (adj.HR per 1-IQR increase 0.65, 95%CI:0.50-0.86; p = 0.002 for BChE and 0.63, 95%CI:0.50-0.79; *p* < 0.001 for albumin). BChE and albumin correlated inversely with CRP (*r* = -0.21, *p* < 0.001 and *r* = -0.36, *p* < 0.001), SAA (*r* = -0.19, *p* < 0.001 and *r* = -0.33, *p* < 0.001) and IL-6 (*r* = -0.13, *p* = 0.009 and *r* = -0.17, *p* = 0.001).

**Conclusions:**

Decreased serum BChE and albumin levels are associated with increased all-cause mortality in treatment-naïve cancer patients without a manifest malignant hepatic involvement irrespective of tumor entity or stage. This association may reflect progressing systemic inflammation and metabolic derangement with subclinical involvement of the liver.

## INTRODUCTION

Liver biomarkers such as alanine aminotransferase (ALT), aspartate aminotransferase (AST), γ-glutamyltransferase (GGT), butyryl-cholinesterase (BChE), albumin and bilirubin are generally used for the clinical diagnosis and estimation of disorders involving liver functional impairment or morphologic liver damage, respectively. Liver parameters have prognostic significance in non-malignant liver diseases [1] but also malignant diseases involving the hepatic system. In hepatocellular carcinoma, GGT was found to be a prognostic marker for survival [2]. Also patients with different primary cancers show deranged liver function tests when suffering from liver metastases, so that the evaluation of AST, ALT and GGT levels has been suggested for liver metastases screening [3, 4].

However, there is evidence that liver parameters have prognostic significance in various clinical conditions of also non-liver origin. Previous studies demonstrated an association between GGT and ALT with the incidence of type II diabetes [5], the occurrence of cardiovascular events [6, 7], and GGT, AST and ALT with all-cause mortality in both the general population and elderly [8–10]. It could therefore be assumed that liver biomarkers could be associated with prognosis in malignant disease generally, even without direct hepatic involvement.

BChE (or pseudocholinesterase) is an α-glycoprotein found in the liver and a solid biochemical marker of liver function. Besides, BChE levels react rapidly to nutritional status as well as clinical conditions associated with inflammation. Another representative of functional liver parameters is albumin, implicated in various conditions including again nutritional status and inflammatory response. Both BChE and albumin have found to be a significant survival factors in terminally ill cancer patients [11]. In a meta-analysis, lower pretreatment albumin levels were found to be related with poor prognosis in gastrointestinal, lung, female and multiple cancers [12]. Similarly, preoperative BChE levels were an independent prognostic factor for overall survival in various cancers of non-liver origin [13–15].

As to now, there are no data regarding the predictive value of liver parameters, especially BChE or albumin, in an unselected cohort of cancer patients. Additionally, no study aimed particularly to differentiate between patients with or without a primary or secondary involvement of the liver for these biomarkers.

The aim of this study was to investigate whether all liver parameters including BChE and albumin have the ability to predict long-term mortality in a treatment naïve cancer patient cohort independently from a malignant hepatic involvement.

## MATERIALS AND METHODS

### Study population

Consecutive patients with a primary diagnosis of cancer were prospectively enrolled at the Vienna General Hospital, a university-affiliated tertiary care center between April 2011 and June 2013. Eligible patients had suspected or confirmed cancer at first presentation and were excluded if they had received any prior anticancer therapy, showed clinical signs of infection or if the diagnosis of cancer could not be confirmed. Patients were classified according to tumor entity and tumor stage. Hepatic involvement was as assessed by searching for reports of different imaging modalities of the liver (i.e. abdominal ultrasound, computed tomography or magnetic resonance) in patients presenting with liver associated malignancies or advanced tumor stages, i.e. patients classified in stages 3 or 4. Comorbidities as hypertension or diabetes mellitus, traditional risk factors as smoking status and medical therapy were recorded. Observation period was 24 months at least. Written, informed consent was obtained from all study participants. The study protocol complies with the Declaration of Helsinki and was approved by the local ethics committee of the Medical University of Vienna (EK 736/2010).

### Laboratory analysis

Venous blood samples were obtained at first hospital presentation and analyzed on-site, according to our local laboratory standard procedures. Routinely available liver enzymes as serum BChE, ALT, AST, and GGT as well as bilirubin and albumin levels were measured. For the interpretation ALT, AST, GGT and bilirubin were referred to as morphological parameters as they can be elevated in liver cell injury, whereas BChE and albumin were termed functional parameters, as their levels are rather depending on the synthetic capacity of the liver. Additionally, the inflammatory markers CRP, SAA and IL-6 were determined.

### Assays

BChE [16], GGT [17], AST [18] and ALT [18] were determined by a kinetic enzyme assay in serum or heparinized plasma. CRP and SAA levels were determined in ethylenediaminetetraacetic acid (EDTA) and heparinized plasma by means of particle enhanced immunonephelometry using the BN II System (Siemens Healthcare Diagnostics, Marburg, Germany). Serum IL-6 was detected with a specific enzyme-linked immunosorbent assay (eBioscience, Vienna, Austria).

### Study endpoint

All-cause mortality was chosen as the primary study endpoint. Data were obtained from the Central Office of Civil Registration Austria.

### Statistical analysis

Continuous data were presented as median and IQR and categorical data as counts and percentages. Medians between groups were compared using the Mann-Whitney-U or Kruskal-Wallis test. The Spearman-Rho correlation coefficient was calculated for the liver parameters and other variables. Cox proportional hazard regression analysis was used to evaluate the effect of liver parameters on all-cause mortality in cancer patients. To account for potential confounding effects, multivariate Cox regression analysis was performed adjusting for a clinical confounder model including age, gender and renal function, and additionally for tumor entity and stage. Results are presented as HRs per IQR. The analysis was carried out in the total patient cohort as well as in the subpopulation of patients without any primary or secondary malignant hepatic involvement. To account for potential gender related differences, Cox regression analysis was performed for both genders separately in the total cohort. To assess the association of BChE and albumin levels with the primary endpoint graphically, the population was divided into tertiles and overall survival for 24 months was presented as Kaplan Meier curves. Groups were compared by the means of the log-rank-test. For all tests two-sided p-values lower 0.05 were considered to indicate statistical significance. The analyses were carried out using the SPSS 22.0 (IBM Corp, New York, NY, USA) and the STATA11 software package (StataCorp, College Station, TX, USA).

## RESULTS

### Baseline characteristics

A total of 555 consecutive patients were enrolled in this prospective cohort study. The detailed baseline characteristics of our study population are displayed in Table 1, a complete description of tumor entities is presented in *Supplemental Table 1*, the distribution of disease states according to tumor entities is displayed in *Supplementary Figure 1*. Median age was 62 (IQR 52-71) and 41% of the patients were male. 33% of patients presented with a tumor stage 4. The median levels of BChE were 7.31kU/l (range 1.16-16.60) and 43.0g/l (range 17.3-64.3) for albumin. BChE and albumin were decreasing with progressive disease state reflected by tumor stage (*Supplemental Table 2*). Twenty-seven (5%) patients were diagnosed with a primary hepatic, or anatomically related, cholangiocellular or pancreatic cancer, whereas another 51 (10%) patients displayed a manifest hepatic involvement by secondary blastomas. A hepatic involvement could definitely be excluded for a total of 453 (82%) patients. For this cohort the described association with tumor stage remained significant for BChE and albumin (*Supplemental Table 3*).

**Table 1:** Baseline characteristics of treatment-naïve patients diagnosed with cancer (*n* = 555)

	Treatment-naïve cancer patients (*n*= 555)
Age, years (IQR)	62 (52-71)
Male gender, n (%)	227 (41%)
BMI kg/m^2^, (IQR)	25.0 (22.6–28.4)
Comorbidities	
Known CAD, *n* (%)	28 (5%)
Heart Failure, *n* (%)	38 (7%)
Diabetes mellitus, *n* (%)	43 (8%)
Arterial Hypertension, *n* (%)	250 (45%)
CKD, *n* (%)	31 (6%)
COPD, *n* (%)	113 (20%)
Cancer disease stage*	
Stage 1, *n* (%)	96 (17%)
Stage 2, *n* (%)	50 (9%)
Stage 3, *n* (%)	108 (19%)
Stage 4, *n* (%)	183 (33%)
Hepatic involvement	
Primary hepatic, biliary tract or pancreatic malignoma, *n* (%)	27 (5%)
Hepatic metastases, *n* (%)	51 (10%)
Laboratory parameters	
GFR, mL/min/1.73 m^2^ (IQR)	74.5 (63.7–86.0)
BUN, mg/dl (IQR)	15 (12-19)
BChE, kU/l (IQR)	7.31 (6.10–8.40)
AST (GOT), U/l (IQR)	24 (19–31)
ALT (GPT), U/l (IQR)	22 (16–32)
GGT, U/l (IQR)	32 (21–63)
Bilirubin, mg/dl (IQR)	0.58 (0.44-0.79)
Albumin, g/l (IQR)	43.0 (40.0–45.4)
CRP, mg/dl (IQR)	0 (0-1)
SAA, μg/ml (IQR)	8 (4-26)
IL-6, pg/ml (IQR)	2 (2–3)

Continuous variables are given as medians and inter-quartile ranges (IQR). Counts are given as numbers and percentages.

IQR – interquartile range; BMI – body mass index, CAD – coronary artery disease; CKD – chronic kidney disease; COPD – chronic obstructive pulmonary disease; GFR – glomerular filtration rate; BUN – blood urea nitrogen; BChE – butyryl-cholinesterase, AST – aspartate transaminase, ALT – alanine transaminase, GGT – gamma glutamyltransferase; CRP - C-reactive protein; SAA - Serum Amyloid A; IL-6 – interleukin 6.

* tumor stage was assessed by the respective treating oncologist and was indicated for all patients excluding those with myeloproliferative neoplasias.

### Correlation of liver parameters and inflammatory markers

In patients without hepatic involvement, the inflammatory markers CRP, SAA and IL-6 displayed a significant inverse correlation with BChE (*r* = -0.21, *p* < 0.001 for CRP, *r* = -0.19, *p* < 0.001 for SAA and *r* = -0.13, *p* = 0.009 for IL-6) and albumin (*r* = -0.36, *p* < 0.001 for CRP, *r* = -0.33, *p* < 0.001 for SAA and *r* = -0.17, *p* = 0.001 for IL-6). Body mass index (BMI) correlated positively with BChE (*r* = 0.26, *p* < 0.001) but not with albumin (*r* = 0.06, *p* = 0.187).

### Survival analysis

186 (34%) patients of the total cohort and 117 (26%) patients without hepatic involvement died during a median follow-up of 25 (IQR 16-31) and 27 (IQR 18-32) months, respectively. The results of the cox regression analysis for the investigated liver parameters are displayed in Table 2 for the total patient cohort, in Table 3 for patients without any hepatic involvement and in Table 4 for patients with hepatic involvement. For the total cohort of cancer patients all liver parameters were significantly associated with all-cause mortality (crude HR per 1-IQR increase 0.51, 95%CI 0.42-0.62 for BChE; 0.55, 95%CI 0.47-0.64 for albumin; 1.11, 95%CI:1.07-1.15 for AST; 1.15, 95%CI:1.08-1.22 for ALT; 1.04, 95%CI:1.02-1.06 for GGT and 1.08, 95%CI:1.05-1.11 for bilirubin; *p* < 0.001 for all) and this association remained significant after adjustment for age, gender, kidney function, tumor entity and tumor stage (adjusted HR per 1-IQR increase 0.55, 95%CI:0.44-0.69 for BChE; 0.64, 95%CI:0.53-0.76 for albumin; 1.12, 95%CI:1.08-1.17 for AST; 1.17 95%CI:1.10-1.24 for ALT; 1.03, 95%CI:1.01-1.05 for GGT and 1.09, 95%CI:1.04-1.13 for bilirubin; *p* < 0.001 for BChE, albumin, AST, ALT and bilirubin and *p* = 0.003 for GGT). In patients without primary or secondary hepatic involvement only the functional parameters BChE and albumin showed a significant association with the primary end-point (crude HR per 1-IQR increase 0.61, 95%CI:0.49-0.77 for BChE; *p* < 0.001 and 0.58, 95%CI:0.47-0.70 for albumin; *p* < 0.001), whereas AST, ALT, GGT and bilirubin were not associated with outcome. This effect remained highly significant after adjustment (adj. HR per 1-IQR increase 0.65, 95%CI:0.50-0.86 for BChE; *p* = 0.002 and 0.63, 95%CI:0.50-0.79 for albumin; *p* < 0.001). Complementary, for patients with a manifest hepatic involvement again all liver parameters revealed a significant association with overall survival (*p* < 0.001 for BChE, albumin, AST and ALT and *p* < 0.05 for GGT and bilirubin). When considering male and female patients separately for the total cohort, BChE, ALT, AST and bilirubin showed different baseline levels. However, all liver parameters except ALT for male patients in the univariate and all liver parameters except GGT for female patients in the adjusted analysis were significantly associated with outcome (Supplementary Table 4). Results remained virtually unchanged when analyzing patients with solely solid tumors (data not shown).

**Table 2:** Unadjusted and adjusted effects of liver morphological and functional parameters on all cause mortality in treatment-naïve cancer patients at the time of first diagnosis (*n* = 555)

Variables	IQR	Crude HR (95%CI)	*P*-value	Adj. HR1 (95%CI)	*P*-value
BChE, kU/l (IQR)	2.30	0.51 (0.42-0.62)	**<0.001**	0.55 (0.44-0.69)	**<0.001**
Albumin, g/l (IQR)	5.4	0.55 (0.47-0.64)	**<0.001**	0.64 (0.53-0.76)	**<0.001**
AST, U/l (IQR)	12	1.11 (1.07-1.15)	**<0.001**	1.12 (1.08-1.17)	**<0.001**
ALT, U/l (IQR)	16	1.15 (1.08-1.22)	**<0.001**	1.17 (1.10-1.24)	**<0.001**
GGT, U/l (IQR)	42	1.04 (1.02-1.06)	**<0.001**	1.03 (1.01-1.05)	**0.003**
Bilirubin, mg/dl (IQR)	0.35	1.08 (1.05-1.11)	**<0.001**	1.09 (1.04-1.13)	**<0.001**

BChE – butyryl-cholinesterase, AST – aspartate transaminase, ALT – alanine transaminase, GGT – gamma glutamyltransferase, IQR – inter quartile range; fonts in bold indicate statistical significance (*p* < 0.05).

Cox proportional hazard models for all liver parameters are shown. Hazard ratios (HR) refer to an increase of one IQR in continuous variables. HRs are adjusted (adj.) for influencing variables, i.e. age, gender, kidney function, tumor entity and tumor stage, respectively.

^1^ HR adjusted to age, gender, kidney function (GFR), tumor stage and tumor entity.

**Table 3:** Unadjusted and adjusted effects of liver morphological and functional parameters on all cause mortality in treatment-naïve cancer patients without hepatic involvement at the time of first diagnosis (*n*=453)

Variables	IQR	Crude HR (95%CI)	*P*-value	Adj. HR1 (95%CI)	*P*-value
BChE, kU/l (IQR)	2.19	0.61 (0.49-0.77)	**<0.001**	0.65 (0.50-0.86)	**0.002**
Albumin, g/l (IQR)	5.4	0.58 (0.47-0.70)	**<0.001**	0.63 (0.50-0.79)	**<0.001**
AST, U/l (IQR)	11	0.94 (0.80-1.09)	0.401	1.02 (0.90-1.16)	0.761
ALT, U/l (IQR)	14	0.95 (0.83-1.06)	0.485	1.02 (0.88-1.21)	0.712
GGT, U/l (IQR)	34	1.03 (0.97-1.06)	0.091	1.02 (0.99-1.06)	0.226
Bilirubin, mg/dl (IQR)	0.34	1.03 (0.90-1.17)	0.704	1.02 (0.87-1.21)	0.777

BChE – butyryl-cholinesterase, AST – aspartate transaminase, ALT – alanine transaminase, GGT – gamma glutamyltransferase, IQR – inter quartile range; fonts in bold indicate statistical significance (*p* < 0.05).

Cox proportional hazard models for all liver parameters are shown. Hazard ratios (HR) refer to an increase of one IQR in continuous variables. HRs are adjusted (adj.) for influencing variables, i.e. age, gender, kidney function, tumor entity and tumor stage, respectively.

^1^ HR adjusted to age, gender, kidney function (GFR), tumor stage and tumor entity.

**Table 4:** Unadjusted and adjusted effects of liver morphological and functional parameters on all cause mortality in treatment-naïve cancer patients with definite hepatic involvement at the time of first diagnosis (*n* = 78)

Variables	IQR	Crude HR (95%CI)	*P*-value	Adj. HR1 (95%CI)	*P*-value
BChE, kU/l (IQR)	2.86	0.41 (0.26-0.65)	**<0.001**	0.41 (0.26-0.64)	**<0.001**
Albumin, g/l (IQR)	4.2	0.71 (0.58-0.87)	**<0.001**	0.71 (0.55-0.91)	**0.007**
AST, U/l (IQR)	27	1.33 (1.17-1.51)	**<0.001**	1.39 (1.21-1.60)	**<0.001**
ALT, U/l (IQR)	33	1.39 (1.19-1.62)	**<0.001**	1.54 (1.30-1.84)	**<0.001**
GGT, U/l (IQR)	136	1.07 (1.00-1.14)	**0.039**	1.07 (1.00-1.15)	**0.047**
Bilirubin, mg/dl (IQR)	0.59	1.07 (1.01-1.13)	**0.015**	1.07 (1.01-1.13)	**0.030**

BChE – butyryl-cholinesterase, AST – aspartate transaminase, ALT – alanine transaminase, GGT – gamma glutamyltransferase, IQR – inter quartile range; fonts in bold indicate statistical significance (p<0.05).

Cox proportional hazard models for all liver parameters are shown. Hazard ratios (HR) refer to an increase of one IQR in continuous variables. HRs are adjusted (adj.) for influencing variables, i.e. age, gender, kidney function, tumor entity and tumor stage, respectively.

^1^ HR adjusted to age, gender and kidney function (GFR).

### Kaplan Meier curves

**Figure 1:**
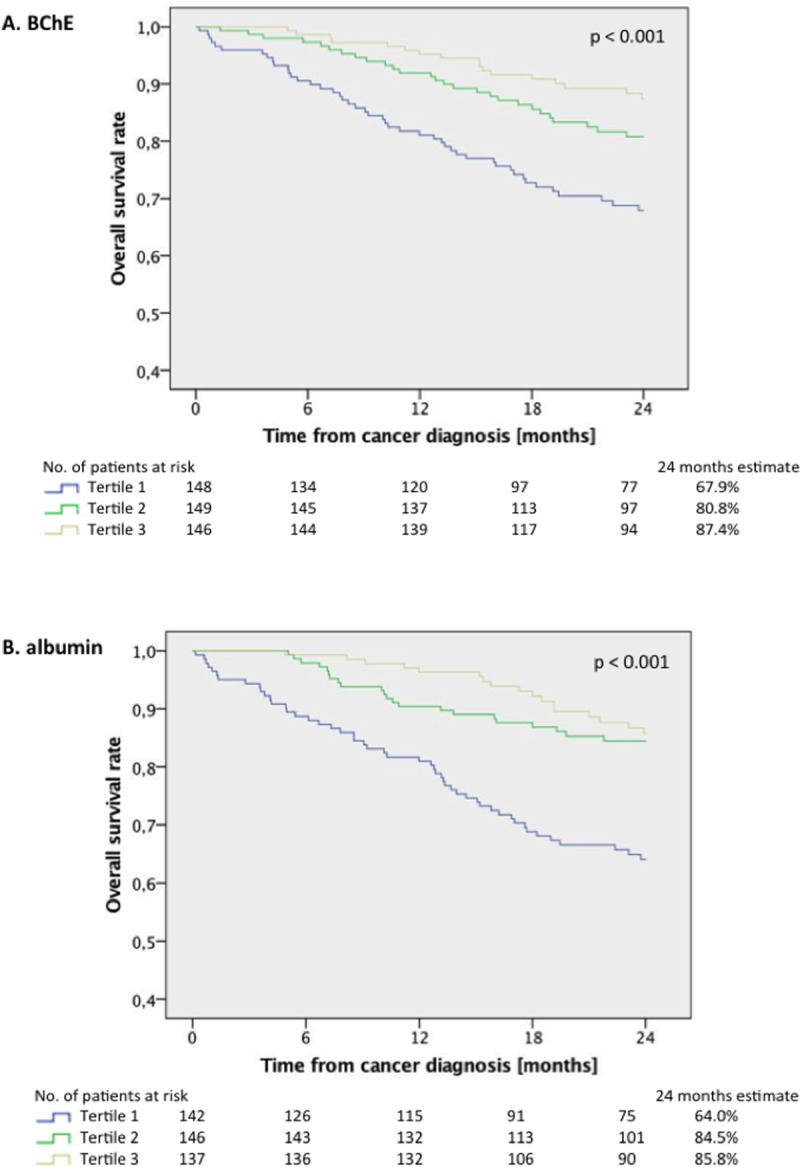
Overall survival rates for treatment-naïve cancer patients without hepatic involvement (*n* = 453) according to tertiles of (**A**) butyryl-cholinesterase (BChE) and (**B**) albumin for a follow-up period of 24 months (*p* < 0.001 between all groups, log-rank test).

Kaplan Meier curves and log-rank analysis (Figure 1) confirmed the high discriminatory power of BChE and albumin on overall survival for patients without hepatic involvement. For BChE the 12 and 24 month estimates were 95.2% and 87.4% in the upper, 91.9% and 80.8% in the mid and 81.1% and 67.9% in the lower tertile (p < 0.001 between all groups). For albumin the 12 months and 24 month estimates were 96.4% and 85.8% in the upper, 90.4% and 84.5% in the mid and 81.0% and 64.0% in the lower tertile (*p* < 0.001 between all groups). For the total observation period the results remained qualitatively unchanged (*p* = 0.002 for BChE and *p* < 0.001 for albumin).

## DISCUSSION

Our study demonstrated that BChE and albumin are robust prognostic factors for overall survival in treatment-naïve cancer patients without manifest hepatic involvement irrespective of tumor entity and stage.

For the total cohort of cancer patients all investigated liver parameters were significantly associated with all-cause mortality. However, this association seems to be driven by malignant hepatic involvement for GGT, AST, ALT and bilirubin, since in patients without primary or secondary hepatic malignancies only the functional parameters BChE and albumin showed a significant association with the primary end-point, which remained significant after multivariate adjustment (adj. HR per 1-IQR increase 0.65, 95%CI:0.50-0.86 for BChE; *p* = 0.002 and 0.63, 95%CI:0.50-0.79 for albumin; *p* < 0.001). Both BChE and albumin displayed a significant negative correlation with the measured inflammatory markers CRP, SAA and IL-6.

### Liver biomarkers in cancer

Liver biomarkers are generally used for the clinical diagnosis and estimation of disorders involving liver functional impairment or morphological damage. Regarding malignant disease, GGT was found to be a prognostic marker for survival in hepatocellular carcinoma [2]. Moreover, the vast majority of patients with liver metastases of various primary cancers of non-liver origin show deranged liver function tests at presentation, so that the evaluation of these biomarkers has been suggested for liver metastases screening [3, 4]. In breast cancer with hepatic metastases, elevated AST and bilirubin were associated with worse prognosis [19]. GGT, AST and ALT were not only shown to be associated with a manifest hepatic malignancy but also with hepatic, and interestingly, overall future cancer incidence [20, 21]. In fact a growing body of evidence indicates that liver biomarkers may comprise prognostic information for various clinical conditions without primary pathological manifestations of the liver [5–10]. Nonetheless, the impact of liver cellular biomarkers specifically on outcome in malignancies without a manifest hepatic involvement has not been investigated yet.

In this study, all liver parameters, i.e. GGT, AST, ALT, BChE, albumin and bilirubin were significant risk predictors for all-cause mortality in the total cancer patient cohort. The association of GGT, AST, ALT and bilirubin with outcome seems to be driven by a manifest primary or secondary malignant involvement of the hepatic system, in line with previous results. However, solely BChE and albumin were highly and significantly associated with the primary endpoint also for patients without a hepatic involvement even after adjustment for age, gender, kidney function, tumor entity and stage, underlining the robust and independent character of these liver biomarkers in cancer generally.

### BChE and albumin in cancer

BChE is a solid biochemical marker of liver organ damage with diminishing plasma BChE activity accompanying a functional breakdown of the liver. Also, BChE levels tend to change rapidly to nutritional status and are closely associated with inflammatory conditions. Decreased levels of BChE have been described in anorexia, as well as in critically ill patients or frail elderly [22]. An inverse correlation of BChE and the inflammatory markers CRP and IL-6 has been reported in a non-cancer cohort [23]. Similarly to other liver enzymes, a surprisingly strong prognostic value of BChE levels have been demonstrated for clinical conditions of non-liver origin as coronary artery disease [24, 25] or cardiac surgery [26], where the role of BChE is not completely understood, however here the reflection of the post surgical host inflammatory response or a general susceptibility by BChE is supposable. Albumin on the other hand is the most commonly monitored hepatic protein in clinical care, since it constitutes almost 50% of plasma proteins in healthy adults. Conditions that affect serum albumin are numerous including alterations in liver function, nutrition and inflammation [27]. In the inflammatory response, the cytokine cascade targets the metabolism of hepatic proteins, with IL-6 being the most important stimulator of acute phase proteins as CRP. Albumin itself is a negative acute phase protein decreasing with acute injury or inflammation and increasing in the recovery phase, independently from nutritional status [27]. Due to its systemic effects, altered albumin levels have not only been implicated in liver diseases, but hypoalbuminemia has also been identified as a risk factor for coronary artery disease [28] and mortality in chronic kidney disease [29]. In summary, BChE and albumin levels should be interpreted in the context of systemic inflammation and malnutrition, both conditions that are hallmarks of malignant disease.

Consequently, BChE and albumin levels have found to be significant survival factors in terminally ill cancer patients [11]. Patients with head and neck or uterine cervix cancer undergoing radiation therapy, showed decreased BChE levels before the induction of therapy, a rise during therapy and resolution to normal levels for patients remaining free of disease at 6 months follow-up [30]. Similarly, in a meta-analysis, lower pretreatment albumin levels were found to be related with poor prognosis in gastrointestinal, lung, female and multiple cancers [12]. In retrospective analyses of clear cell renal carcinoma patients, invasive bladder cancer and prostate cancer, preoperative BChE levels were an independent prognostic factor for overall survival [13–15]. Moreover, nutritional support in cancer patients resulted in augmented BChE levels parallel to an increase in body weight [31].

The negative correlation of BChE and albumin with the inflammatory markers in this study supports previous observations for cancer patients. In contrast, our data revealed no correlation of albumin with nutritional status reflected by BMI. Albumin synthesis can be rate limited, yet this is only seen rarely in extreme malnutrition [32], which does not apply for our cohort since the BMI with a median of 25.0 (IQR 22.6-28.4) kg/m^2^ was normal. For cancer patients an inverse relation between BMI and albumin is described, which has been attributed to an enhanced compensatory synthesis [12]. However, in later stages, albumin synthesis is suppressed by inflammation and malnutrition [12]. We could speculate that in malignant disease albumin is a sensitive parameter responding rather early to systemic inflammation without strict correlation to nutritional status. In opposition to albumin, BChE not only showed a significant correlation with inflammatory status but also with BMI, in line with the literature [23, 33]. In accordance to previous studies, we found decreasing BChE and albumin levels with progressing tumor stage for both the total cohort and patients without hepatic involvement. In addition, our study extends the prognostic value of plasma BChE and albumin for all-cause mortality in an unselected treatment-naïve cancer patient cohort irrespective of tumor entity or stage.

### Inflammation, malnutrition and cancer prognosis

Malignancy is a systemic disease involving both inflammation and a disrupted energy metabolism, whereas the two conditions are strongly linked up. [34, 35] Although malignant disease strongly relies on genetic and environmental factors, it seems evident that host inflammatory status and response are equally crucial for the development and progression of the disease. By time it also became clear, that the systemic inflammatory response plays an important role in the patients nutritional and functional decline, as much that CRP has been added to the definition of cancer cachexia [36]. Emphasizing the clinical importance of this pathophysiologic relation, C-reactive protein (CRP) and albumin have been selected from an extensive range of laboratory parameters to build a prognostic score termed modified Glasgow Prognostic Score, which has widely been validated in cancer patients since the initial description [37, 38].

Given its anatomical location and specific structure the liver homes numerous immune cells and can be regarded as part of the immune system participating in both adaptive and non-specific inflammatory response of the organism [39]. Immuno-incompetence and malnutrition in patients with liver disease is a well known phenomenon [40]. Liver cirrhosis for example is accompanied by an acquired immune deficiency and systemic inflammation based on enhanced translocation of pathogens, stimulation of circulating immune cells by damage-associated molecular patterns and an imbalance between pro- and anti-inflammatory processes [41]. The accumulation of lymphocytes and their modulation by cytokines and immunoglobulins are involved in the specific response, which includes immune surveillance and first line defenses against pathogens from the gastrointestinal tract. However, probably more important, is the contribution of the liver to the acute phase response. The liver is the predominant target organ for cytokines originating from a broad range of cells, at distant inflammatory processes with a crucial role of the cellular immune system, whereas IL-6 is the principal mediator of acute phase protein synthesis [42]. Acute phase proteins are thought to aim to restore homeostasis by complex effects as activation of complement, inhibition of cell growth or ease the elimination of targeted cells [43, 44]. However, acute phase responses may not uniformly be beneficial and persisting acute phase response as in cancer may result in metabolic disturbances, cachexia and reduced response to treatment. Modulating the inflammatory response could be an option for optimizing nutrition in patients with malignant diseases [45].

In this context, BChE and albumin are remarkably interesting biomarkers arising from the central organ responsible for both inflammatory response and energy homeostasis. BChE could as well as be another marker for the extent of systemic inflammation, and based on its more robust association with nutritional status, also a more sensitive indicator of nutritional decline associated with worse prognosis in cancer patients. The inclusion of unselected patients with various types of cancer enables a rather unbiased approach for the investigation of liver parameters especially BchE and albumin underlining the relevance of this clinical finding.

### Remote organ response to malignant disease

Our group recently reported, that cancer also seems to be able to influence the neurohumoral system with elevated cardiac biomarkers in the absence of a manifest cardiac disease, in terms of a subclinical involvement [46]. According to this study, very similar notions apply for the liver and assumedly for other, if not all, organ systems. Regarding the hepatic system, all biomarkers react to as a rough affection as a primary or secondary malignant involvement. After exclusion of the patients with direct affection of the liver however, only the probably more sensitive functional markers BChE and albumin seem to indicate disease severity, whereas the cellular markers AST, ALT and GGT indicating direct liver damage stay behind. We therefore have to come to consider malignancy as a systemic disease generally disrupting the prevalent subtle physiologic homeostasis probably by the induction of a systemic inflammatory process. The respective organ systems may sense cancer affection responding by partly modest alterations of the most sensitive functional markers.

## LIMITATIONS

Even though the total number of patients enrolled into this analysis is relatively high, a larger-scaled study might still be needed to validate the present data. Also specific differences between various types of cancer might be revealed by further studies investigating just one type of malignancy. Laboratory measurements have been performed only at a single time point prior to initiation of anticancer therapy and studies with serial measurements throughout disease progression might provide additional insights. Finally, while our endpoint is all-cause mortality, precise information about the percentage of cancer-related death would certainly be of important clinical interest. However, sincepost hoc interpretations of certifications of death are not reliable, the development of a cardiac disease during cancer progression should be documented in longitudinal studies in the future.

## CONCLUSIONS

The data clearly show that plasma levels of BChE and albumin are powerful predictors for all-cause mortality in an unselected treatment-naïve cancer patient cohort without manifest hepatic involvement irrespective of tumor type or stage. This association is probably based on an interplay between systemic inflammation and metabolic derangement progressing with cancer disease severity mirrored by functional parameters of the liver, which is the central organ for both energy homeostasis and inflammatory response.

## SUPPLEMENTARY MATERIALS FIGURES AND TABLES





## Abbreviations

ALT: alanine aminotransferase
AST: aspartate aminotransferase
GGT: γ-glutamyltransferase
BChE: butyryl-cholinesterase
CRP: C-reactive protein
SAA: serum amyloid A
IL-6: interleukin-6
mGPS: modified Glasgow Prognostic Score
EDTA: ethylenediaminetetraacetic acid
NRI: net reclassification index
ROC: receiver operator characteristics
HR: hazard ratio
IQR: inter-quartile range
