# Supplementary material for: Subclinical involvement of the liver is associated with prognosis in treatment naïve cancer patients
Source: Oncotarget. 2017 Apr 16;8(46):81250–60. doi: 10.18632/oncotarget.17131 (PMC5655279; doi:10.18632/oncotarget.17131)
Supplement: Supplementary file 1 [file oncotarget-08-81250-s001.pdf]

Subclinical involvement of the liver is associated with prognosis in treatment naïve cancer patients

Supplementary Material

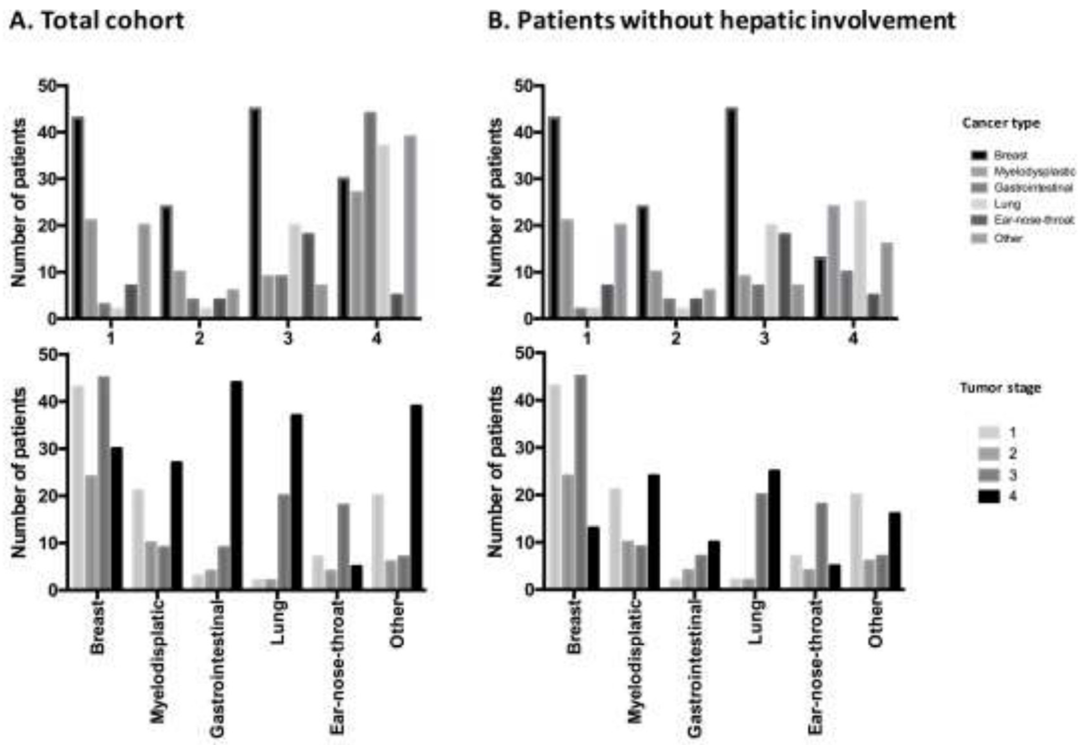

Supplementary Figure 1: Disease stage distribution for treatment-naïve cancer patients. A. shows different tumor stages according to cancer type and cancer types according to tumor stages for the total cohort and B. for patients without hepatic involvement.

For Supplementary Tables see in Supplementary Files
