# Supplementary material for: Subclinical involvement of the liver is associated with prognosis in treatment naïve cancer patients
Source: Oncotarget. 2017 Apr 16;8(46):81250–60. doi: 10.18632/oncotarget.17131 (PMC5655279; doi:10.18632/oncotarget.17131)
Supplement: Supplementary file 2 [file oncotarget-08-81250-s002.docx]

**Supplementary Table 1:** Tumor entities of treatment-naïve cancer patients (n=555). Counts are given as numbers and percentages.

|  | **Cancer patients (n=555)** |
| --- | --- |
| **Tumor entity** |  |
| Lung cancer, n (%) | 61 (11.0%) |
| Breast cancer, n (%) | 146 (26.3%) |
| Brain tumor, n (%) | 23 (4.1%) |
| ENT-tumor, n (%) | 33 (5.9%) |
| Gastrointestinal tumors, n (%) | 67 (12.1%) |
| Myelodysplastic malignancies, n (%) | 68 (12.3%) |
| Myeloproliferative neoplasias, n (%) | 99 (17.8%) |
| Esophageal cancer, n (%) | 11 (2.0%) |
| Testicular cancer, n (%) | 2 (0.4%) |
| Neuroendocrine tumor, n (%) | 11 (2.0%) |
| Sarcoma, n (%) | 9 (1.6%) |
| Mesothelioma, n (%) | 3 (0.6%) |
| Prostate cancer, n (%) | 2 (0.4%) |
| Renal cell carcinoma, n (%) | 4 (0.7%) |
| Thymoma, n (%) | 1 (0.2%) |
| Skin cancer, n (%) | 2 (0.4%) |
| Urogenital tumors, n (%) | 2 (0.4%) |
| Oral cancer, n (%) | 1 (0.2%) |
| Other, n (%) | 10 (1.8%) |

ENT – ear, nose, throat; Myelodysplastic malignancies – haematological malignancies with abnormal differentiation of myeloid or lymphoid cell lines (e.g. AML, ALL, lymphomas, multiple myeloma); Myeloproliferative neoplasias – haematological neoplasias with normal cell differentiation (e.g. essential thrombocytosis, polycythemia vera, myelofibrosis).

**Supplementary Table 2: Butyryl-cholinesterase and albumin levels for the unselected treatment-naïve patient cohort with newly diagnosed cancer according tumor stage (n=555).**

|  | **Stage 1**  **(n=96)** | **Stage 2**  **(n=50)** | **Stage 3**  **(n=108)** | **Stage 4**  **(n=183)** | **p-value** |
| --- | --- | --- | --- | --- | --- |
| BChE, kU/l (IQR) | 7.76 (6.55-9.09) | 7.50 (6.55-8.50) | 7.39 (6.31-8.35) | 6.94 (5.62-8.06) | **<0.001** |
| Albumin, g/l (IQR) | 44.25 (41.10-46.30) | 43.55 (41.10.-45.70) | 43.10 (40.30-45.65) | 41.30 (37.80-44.05) | **<0.001** |

BChE – butyryl-cholinesterase, IQR – inter quartile range.

Continuous variables are given as medians and inter-quartile ranges (IQR), counts are given as numbers and percentages. Variables were compared by the means of the Kruskal-Wallis-test. Fonts in bold indicate statistical significance (p<0.05).

**Supplementary Table 3: Butyryl-cholinesterase and albumin levels for the unselected treatment-naïve patient cohort with newly diagnosed cancer in the absence of hepatic involvement according tumor stage (n=453).**

|  | **Stage 1**  **(n=95)** | **Stage 2**  **(n=50)** | **Stage 3**  **(n=106)** | **Stage 4**  **(n=94)** | **p-value** |
| --- | --- | --- | --- | --- | --- |
| BChE, kU/l (IQR) | 7.80 (6.57-9.11) | 7.50 (6.55-8.50) | 7.41 (6.37-8.38) | 7.19 (5.65-8.06) | **0.013** |
| Albumin, g/l (IQR) | 44.30 (41.30-46.30) | 43.55 (41.10-45.70) | 43.10 (40.30-45.70) | 40.85 (37.30-43.80) | **<0.001** |

BChE – butyryl-cholinesterase, IQR – inter quartile range.

Continuous variables are given as medians and inter-quartile ranges (IQR), counts are given as numbers and percentages. Variables were compared by the means of the Kruskal-Wallis-test. Fonts in bold indicate statistical significance (p<0.05).

**Supplementary Table 4: Liver parameters and their influence on overall survival for treatment-naïve cancer patients according to gender (n=555).**

|  | Total cohort (n=555) | | |  | Male (n=227) | | | | Female (n=328) | | | |  |
| --- | --- | --- | --- | --- | --- | --- | --- | --- | --- | --- | --- | --- | --- |
| Variables | male | female | P-value | IQR  (total cohort) | Crude HR (95%CI) | P-value | Adj. HR^1^ (95%CI) | P-value | Crude HR (95%CI) | P-value | Adj. HR^1^ (95%CI) | P-value | |
| BChE, kU/l (IQR) | 6.99 (5.67-8.35) | 7.48 (6.36-8.52) | **0.005** | 2.30 | 0.59 (0.45-0.76) | **<0.001** | 0.63 (0.47-0.84) | **0.002** | 0.46 (0.34-0.62) | **<0.001** | 0.47 (0.33-0.65) | **<0.001** | |
| Albumin, g/l (IQR) | 43.0 (39.5-45.5) | 43.1 (40.3-45.3) | 0.970 | 5.4 | 0.59 (0.47-0.74) | **<0.001** | 0.63 (0.49-0.81) | **<0.001** | 0.52 (0.42-0.64) | **<0.001** | 0.54 (0.42-0.71) | **<0.001** | |
| AST, U/l (IQR) | 24 (20-34) | 23 (19-30) | 0.087 | 12 | 1.22 (1.13-1.31) | **<0.001** | 1.19 (1.10-1.28) | **<0.001** | 1.10 (1.05-1.15) | **<0.001** | 1.12 (1.06-1.19) | **<0.001** | |
| ALT, U/l (IQR) | 24 (18-38) | 21 (16-29) | **<0.001** | 16 | 1.10 (0.99-1.22) | 0.080 | 1.14 (1.03-1.27) | **0.011** | 1.17 (1.09-1.27) | **<0.001** | 1.19 (1.09-1.30) | **<0.001** | |
| GGT, U/l (IQR) | 39 (26-80) | 28 (18-50) | **<0.001** | 42 | 1.04 (1.02-1.06) | **<0.001** | 1.03 (1.02-1.05) | **0.003** | 1.04 (1.01-1.07) | **0.006** | 1.03 (0.99-1.07) | 0.130 | |
| Bilirubin, mg/dl (IQR) | 0.63 (0.49-0.94) | 0.55 (0.41-0.72) | **<0.001** | 0.35 | 1.09 (1.00-1.19) | **0.045** | 1.12 (1.02-1.24) | **0.024** | 1.07 (1.04-1.11) | **<0.001** | 1.08 (1.03-1.13) | **0.002** | |

BChE – butyryl-cholinesterase, AST – aspartate transaminase, ALT – alanine transaminase, GGT – gamma glutamyltransferase, IQR – inter quartile range.

Variables are given as median and inter-quartile range (IQR), medians between groups were compared using the Mann-Whitney-U test. Cox proportional hazard models for all liver parameters are shown. Hazard ratios (HR) refer to an increase of one IQR of the total cohort in continuous variables as indicated. Fonts in bold indicate statistical significance (p<0.05).

^1^ HR adjusted to age, kidney function (GFR) and tumor stage.
